# The energy conservation and emission reduction co-benefits of China’s emission trading system

**DOI:** 10.1038/s41598-023-40811-4

**Published:** 2023-08-23

**Authors:** Chenggang Li, Ziling Chen, Yongxiang Hu, Chengcheng Cai, Xintong Zuo, Guofei Shang, Hongwei Lin

**Affiliations:** 1 School of Big Data Application and Economics, Guizhou University of Finance and Economics, Guiyang, 550025 Guizhou China; 2 Guizhou Key Laboratory of Big Data Statistical Analysis, Guizhou University of Finance and Economics, Guiyang, 550025 Guizhou China; 3 New Structure Financial Research Center, Guizhou University of Finance and Economics, Guiyang, 550025 Guizhou China; 4 Hebei International Joint Research Center for Remote Sensing of Agricultural Drought Monitoring / School of Land Science and Space Planning, Hebei GEO University, Shijiazhuang, 050031 China; 5 School of Critical Studies, University of Glasgow, Glasgow, G12 8QQ Scotland, UK; 6 School of Public Health, Hubei University of Medicine, Shiyan, 442000 China

**Keywords:** Conservation biology, Environmental social sciences, Mathematics and computing

## Abstract

Emission Trading System (ETS) is an innovative practice under the progress of green development in China. It is also an important method for China to achieve market-oriented environmental governance in ecological civilization construction. The ETS pilot policy has implemented for more than 10 years. However, the co-benefits of ETS pilot policy by the integration of energy consumption, carbon and sulfur dioxide emissions, and wastewater has not been evaluated. In order to fill this gap, we use the 2003–2017 annual data of 30 China’s provinces (municipalities and autonomous regions), and utilize the Difference-in-Differences (DID) model and Propensity Score Matching (PSM-DID) methodology to evaluate the co-benefits of ETS pilot policy on energy conservation and emission reduction. We find that the ETS pilot policy significantly promote energy conservation and emission reduction. Eastern and central China have significantly benefited from the policy, while the western China has not due to the limited technology and innovation as well as an imbalance of the industrial structure. The results provide the policy reference for China’s government and institutions as well as the governments and institutions around the world to fulfill their commitments to save energy and reduce emissions, and early achieve the carbon peaking and carbon neutralization.

## Introduction

The *World Energy Statistics Yearbook* (2019) pointed out that the growth rate of carbon emissions generated in the process of global energy consumption and energy use has reached the highest level since 2010. Furthermore, the Institute of Health Effects, which issued the State of the *World Air 2019 Report*, claimed that the number of people who died of stroke, heart disease, lung cancer, diabetes, and chronic lung disease worldwide in 2017 reached nearly 5 million due to long-term outdoor and indoor air pollution. Many health and ecological issues that humans currently face are closely related to energy consumption and pollution emissions. On September 15, 2020, the Chinese President Xi made an important commitment at the *75th United Nations General Assembly*, emphasizing that China strives to reach the peak of carbon dioxide emissions by 2030 and strives to achieve carbon neutrality by 2060. However, based on data from the *BP World Energy Statistical Yearbook 2019*, it can be found that China’s coal production and coal consumption in 2018 accounted for 46.0% and 50.5% of the world’s total respectively. Economic growth, population structure, and urbanization inevitably increase energy pressure^1^. The Emission Trading System (ETS) is an innovative practice under the background of China’s efforts to promote green development. The connotation of the ETS is to use the market mechanism to establish a legal pollutant emission right that is allowed to be bought and sold like commodities. More importantly, it controls pollutant emissions so as to help achieve the co-benefit purpose of reducing emissions and protecting the environment. Using the ETS pilot policy, we hope to reduce energy consumption and greenhouse gas emissions through market mechanisms, and ultimately achieve the goal of energy conservation and emission reduction.

Regarding the existing researches on the ETS, some scholars only evaluated the role and effectiveness of ETS^2–6^, such as the impact on enterprise production, foreign investment and price fluctuation^7–11^. Some studies only evaluate the role of ETS from the perspective of energy conservation, to promote the achievement of energy conservation and emission reduction goals^12–15^. Other scholars explored the energy conservation and emission reduction of different energy sources^16,17^, and further discussed the impact mechanism of ETS on energy^18^, and the spatial spillover effect on environmental pollution^19,20^. In addition, there are some scholars who only studied the impact of ETS on emission reduction, for example, the effectiveness of ETS is explored from the emission of pollutants such as SO2, CO2 and PM2.5^21–24^. Of course, a small number of scholars began to study the relationship between ETS and energy conservation and emission reduction, and analyzed the energy conservation and emission reduction effects of different industries^25–27^.

By reviewing the above literature, we can find that most scholars tend to study only the single impact of ETS on energy conservation and emission reduction, especially more on energy conservation. However, little attention has been paid to the energy conservation and emission reduction co-benefit of ETS policy. And few researchers pay attention to the policy effects of the ETS from the perspective of energy consumption in China. What’s more, the ETS is regarded as an effective and efficient way to push enterprises to upgrade industry and innovate technology by limiting the total quantity of pollution emissions and changing energy consumption. On the one hand, there are limited empirical explorations of the casual relationship between the ETS policy and energy conservation and emission reduction. We conduct empirical research on the co-benefit of ETS policy on energy conservation and emission reduction, which makes up for the shortcomings of existing research. On the other hand, previous studies on pollutants to evaluate the emission reduction effect mostly focused on a single pollutant, which was not comprehensive enough. We selected four pollutant emissions for research and enriched the relevant research content.

In order to fill the research gap and enrich the research of ETS effect evaluation, differ from the above research, we consider the energy consumption, sulfur dioxide emissions and wastewater and other factors to evaluate the co-benefit of ETS policy. We try to solve the following two questions. First, whether ETS can significantly boost the energy conservation and emission reduction at the same time? Second, whether the effect of ETS on energy conservation and emission reduction in different regions is heterogeneous? The solution to these problems is very important to China’s commitment to achieving carbon peak and carbon neutrality, and is conducive to promoting high-quality economic development.

**Figure 1:**
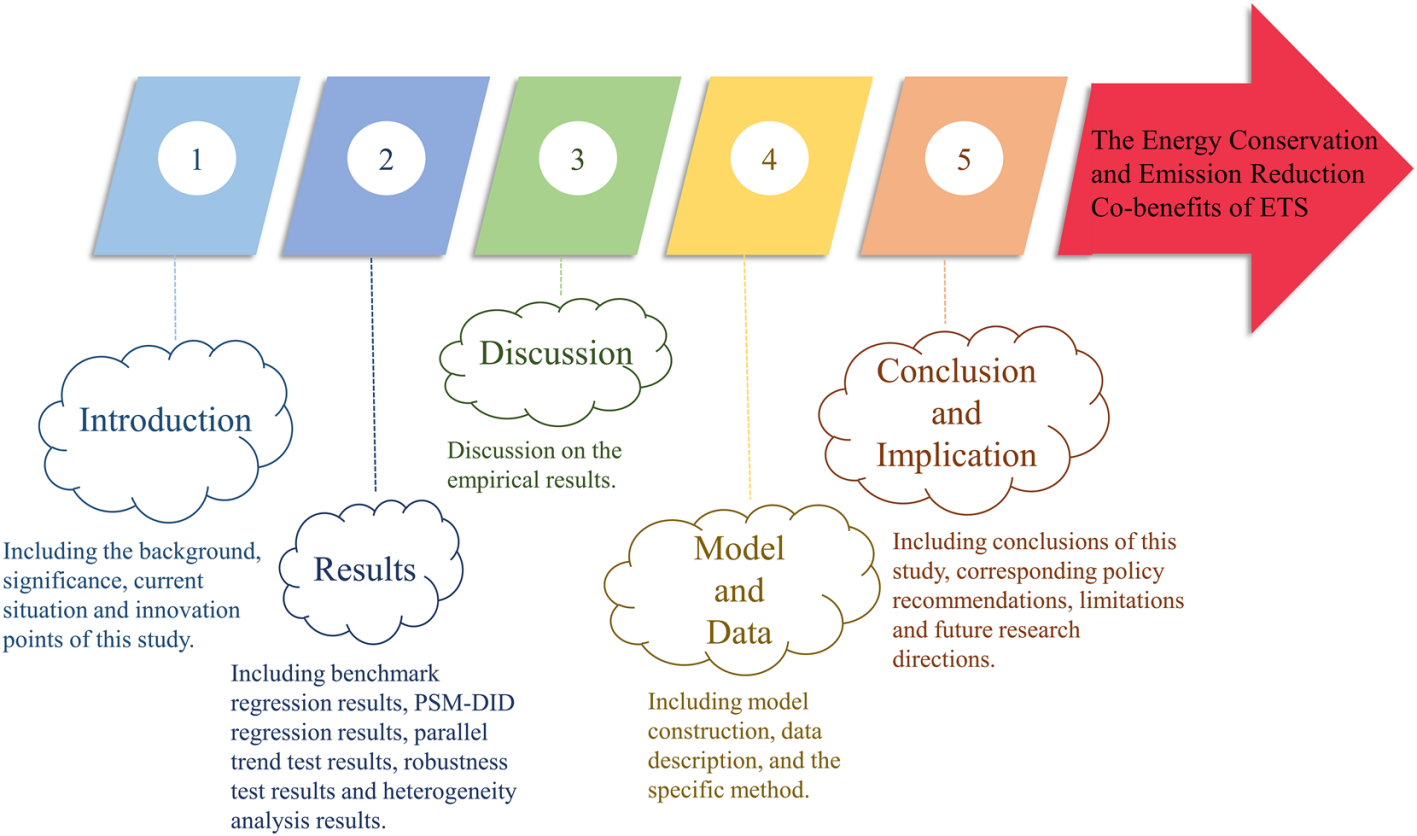
The flow chart of this study.

In this study, we use the Difference-in-differences (DID) to study the energy conservation and emission reduction co-benefits of ETS and use Propensity Score Matching (PSM) to eliminate the influence of provincial and municipal characteristics, and enhance the accuracy and rigor of the model. The flow chart of this study is shown in Fig. 1, and the main tasks of this study are as follows. First, the total index of the explained variable is constructed using the entropy method. Second, we use the DID model and PSM-DID model to evaluate the co-benefits of ETS on the energy conservation and emission reduction of China’s 30 provinces, municipalities and autonomous regions (excluding Tibet, Hong Kong, Macao and Taiwan). Third, we conduct a heterogeneous policy evaluation on the ETS, and provide policy recommendations for balancing the policy effects in China.

## Model and data

### Model

In order to investigate whether the ETS promotes energy conservation and emission reduction co-benefits, we regard the emission trading system pilot as a quasi-natural experiment to analyze its co-benefits. We determine the control group and the treatment group before model construction. In 2007, the Chinese government approved the pilot ETS in 11 regions. This is a major and fundamental institutional innovation and reform in the field of environmental resources in China, and it is the first large-scale pilot work to carry out paid use and trading of pollutant discharge rights. Although relevant policies have been followed up in the following years, their influence and effect are not representative enough.

**Figure 2:**
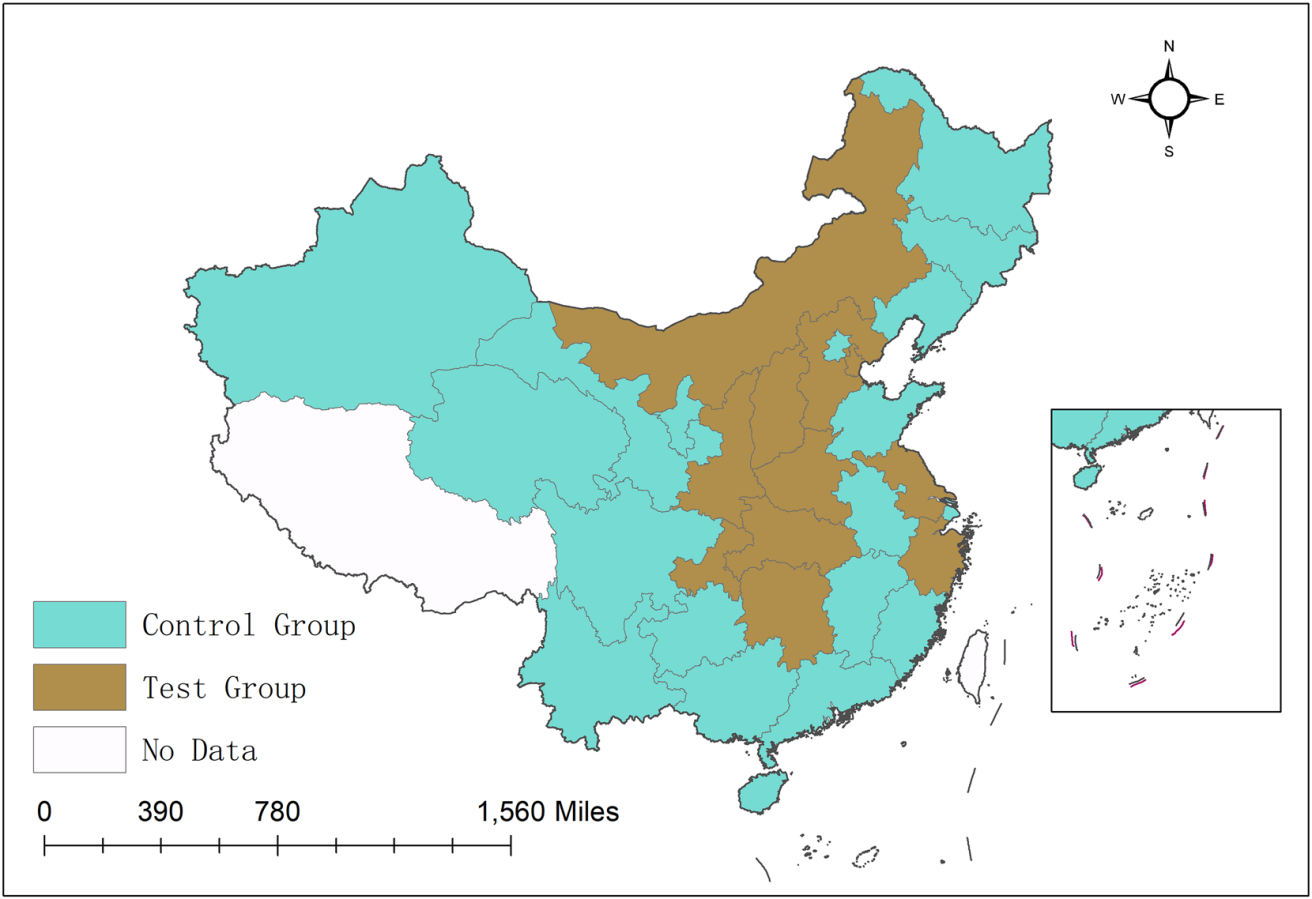
Spatial distribution of the policy pilot groups. The brown legend indicates the spatial distribution of the treatment group, and the green legend indicates the spatial distribution of the control group.

The specific policy pilot regions and the spatial distribution of the policy pilot groups are shown in Fig. 2. On one hand, there are two groups presented in Table 1 and Fig. 2, that is, the treatment group and the control group. Specifically, the treatment groups are the places where the ETS policy is piloted, while the control groups are the provinces, municipality and autonomous region where the ETS policy is not piloted. On the other hand, the region is divided into three parts, namely, eastern, central, and western China. As we can see from the treatment groups, there are 3 provinces and 1 municipality in eastern China that the ETS policy is piloted. They are Tianjin, Hebei, Jiangsu, and Zhejiang. There are 4 regions in the central China, that is, Shanxi, Henan, Hubei, and Hunan. There are 3 regions in the western China, that is, the Inner Mongolia, Chongqing, and Shanxi. According to the distribution of policy pilot areas, we know that the distribution of the policy pilot areas is relatively balanced, and the geographical choices imply that the economic and policy advantages are the primary considerations for pilot policies.

**Table 1:** Specific policy pilot groups.

	Treatment group	Control group
Eastern region	Tianjin, Hebei, Jiangsu, Zhejiang	Beijing, Liaoning, Jilin, Heilongjiang, Shanghai, Fujian, Shandong, Guangdong, Hainan
Central region	Shanxi, Henan, Hubei, Hunan	Anhui, Jiangxi
Western region	Inner Mongolia, Chongqing, Shaanxi	Guangxi, Sichuan, Guizhou, Yunnan, Gansu, Qinghai, Ningxia, Xinjiang

We set 11 policy piloted regions as the experimental groups, and the remaining 19 regions as the control groups. The samples exclude Tibet, Hong Kong, Macao and Taiwan due to the data quality issues and unavailability. By setting 2007 as the year of policy implementation, we use DID to investigate the impact of the ETS policy on the energy conservation and emission reduction co-benefit. The model setting is shown in Formula (1).

1 $$ ECER_{it} = \beta_{0} + \beta_{1} Time_{{\text{t}}} + \beta_{2} Treat_{i} + \beta_{3} PWQ_{it} + \beta_{4} X_{it} + \varepsilon_{it} , $$

where, $ECER_{it}$ is the energy use and pollution emission level of the i-th province (municipality or autonomous region) at the t-th year. $Time_{t}$ means that the ETS policy is piloted at the t-th year. Its value is assigned to 1 after the ETS policy is piloted, while its value is assigned to 0 before the ETS policy is piloted. We set t to be 2007. $Treat_{i}$ indicates whether the i-th province (municipality or autonomous region) belongs to the treatment group or the control group. If the i-th province (municipality or autonomous region) is in the treatment group, the value is assigned to 1. If the i-th province (municipality or autonomous region) is in the control group, the value is assigned to 0. ${\text{PWQ}}_{it}$ is the policy co-benefit of the ETS. If the ETS policy is implemented in the i-th province (municipality or autonomous region), it is assigned to 1 after the year of 2007, otherwise it will be assigned to 0 in 2006 and before. $X_{it}$ represents the control variables. $\varepsilon_{it}$ represents the error term.

### Variables

#### Explained variable

We draw on the research ideas of Shi et al.^28^, Zhang and Ma^29^ to construct the total index level of energy use and pollution emissions (ECER index). The specific indicator connotations are shown in Table 2. We use the entropy method to assign weights to 7 indicators, calculate the ECER index, and finally obtain the ECER data of 30 provinces (municipalities and autonomous regions).

**Table 2:** ECER indicator system.

First level indicators	Second level indicators	Third level indicators	Unit	Attribute
Energy use (EC)	Energy consumption	Total energy consumption	10,000 tons of standard coal	–
		Energy consumption intensity	Tons of standard coal/ten thousand yuan	–
	Resource consumption	Total water consumption	Ten thousand tons	–
Pollutant emissions (ER)	Emission amount	Total wastewater discharge	One hundred million cubic meters	–
		Sulfur dioxide emissions	Ten thousand tons	–
		Ammonia nitrogen emissions in wastewater	Ten thousand tons	–
		COD emissions in wastewater	Ten thousand tons	–

#### Core explanatory variable

The core explanatory variable of this article is the policy effect of the ETS (*PWQ*). It can be seen from Formula (1) that, if $\beta_{3}$ is negative and statistically significant, it indicates that the ETS can promote energy conservation and emission reduction, while if not, it has an inhibiting effect.

#### Control variables

Referring to the method of Lin and Tan^30^, we chose per capita GDP (RG) to measure the level of economic development of all provinces, municipalities and autonomous regions, and perform logarithmic processing on per capita GDP data. Referring to the research of Liu and Chen^31^, we select population density (*RK*) to measure the degree of population consumption of resources and the environment. Referring to the method of Fu et al.^16^, due to the limitation of the data availability, we chose pollution control investment (*ZW*) to measure the governance level. More specifically, pollution control investment is expressed as the ratio of investment in industrial pollution control to GDP. Therefore, we chose per capita GDP, population density and pollution control investment as the control variables.

### Data

We finally select the annual data of 30 provinces, municipalities and autonomous regions (excluding Tibet, Hong Kong, Macao and Taiwan) from 2003 to 2017, and use interpolation to complete the data for the small amount of missing data. The data comes from the *China Energy Statistical Yearbook* (https://data.cnki.net/yearBook), the *Ministry of Environmental Protection of China* (https://www.mee.gov.cn/) and the *National Bureau of Statistics of China* (http://www.stats.gov.cn/).

## Results

### Descriptive statistics

Due to the serious lack of data in some cities, this study finally selects the annual data of 30 provinces and cities (excluding Tibet, Hong Kong, Macao and Taiwan) from 2003 to 2017, and uses the interpolation method to complete the data for a small amount of missing data. The descriptive statistics of each variable are shown in Table 3. The variable ECER has a large mean and a small standard deviation, indicating that the overall level of energy use and pollution emissions is high and relatively balanced.

**Table 3:** Results of descriptive statistics.

Variables	Symbols	Sample capacity	Mean value	Maximum	Minimum	Standard deviation
Level of total indicators of energy use and pollution emissions	ECER	450	0.5146	0.8184	0.1565	0.1234
Policy effects of emission trading system	PWQ	450	0.2689	1	0	0.4439
Regional effect	Treat	450	0.3667	1	0	0.4824
Time effect	Time	450	0.2689	1	0	0.4439
GDP per capita	RG	450	10.2292	11.7675	8.1895	0.7283
Density of population	RK	450	7.6644	8.7495	5.2257	0.6458
Investment in pollution control	ZW	450	1.3379	4.24	0.3	0.6692

### Co-benefits of ETS policy

China’s ETS policy brings the energy conservation and emission reduction co-benefits and the ETS is an effective and efficient policy to promote energy conservation and emission reduction (Table 4). The results of DID model and DID-PSM model show that whether control variables are added or not, the regression coefficient of the core explanatory variable (*PWQ*) is significantly negative (*β*_3, Model (1)_ =  − 0.0930, *P* < 0.01; *β*_3, Model (2)_ =  − 0.1010, *P* < 0.1; *β*_3, Model (3)_ =  − 0.0877, *P* < 0.05; *β*_3, Model (4)_ =  − 0.0961, *P* < 0.01). The ETS policy significantly reduces energy use and pollution emissions after the policy is piloted. The regression results of Model (2) and Model (4) also show that per capita GDP, pollution control investment and population density promote energy conservation and emission reduction.

**Table 4:** Impact of PWQ on ECER.

Variables	DID model		DID-PSM model	
	Model (1)	Model (2)	Model (3)	Model (4)
*PWQ*	− 0.0930*** (0.152)	− 0.1010* (0.195)	− 0.0877** (0.170)	− 0.0961*** (0.163)
*RG*		0.1971* (0.302)		0.0961 (0.255)
*RK*		0.0250 (0.152)		0.0463* (0.137)
*ZW*		0.0760* (0.176)		0.0872*** (0.123)
*_Cons*	0.4180*** (0.145)	− 2.0360* (0.986)	0.455*** (0.156)	− 0.851 (0.793)
Time effect	Yes	Yes	Yes	Yes
Region effect	Yes	Yes	Yes	Yes
*R*^*2*^	0.3012	0.4165	0.3997	0.5192
*N*	450	450	200	200

(1) Model (1) and (3) represent the regression results without control variables, and Model (2) and (4) represent the regression results with control variables. (2) The data in brackets are standard deviation values. (3) *, ** and *** represent the significance at the significance level of 10%, 5% and 1%, respectively. The regression results pass the parallel trend test.

### Robustness test

**Figure 3:**
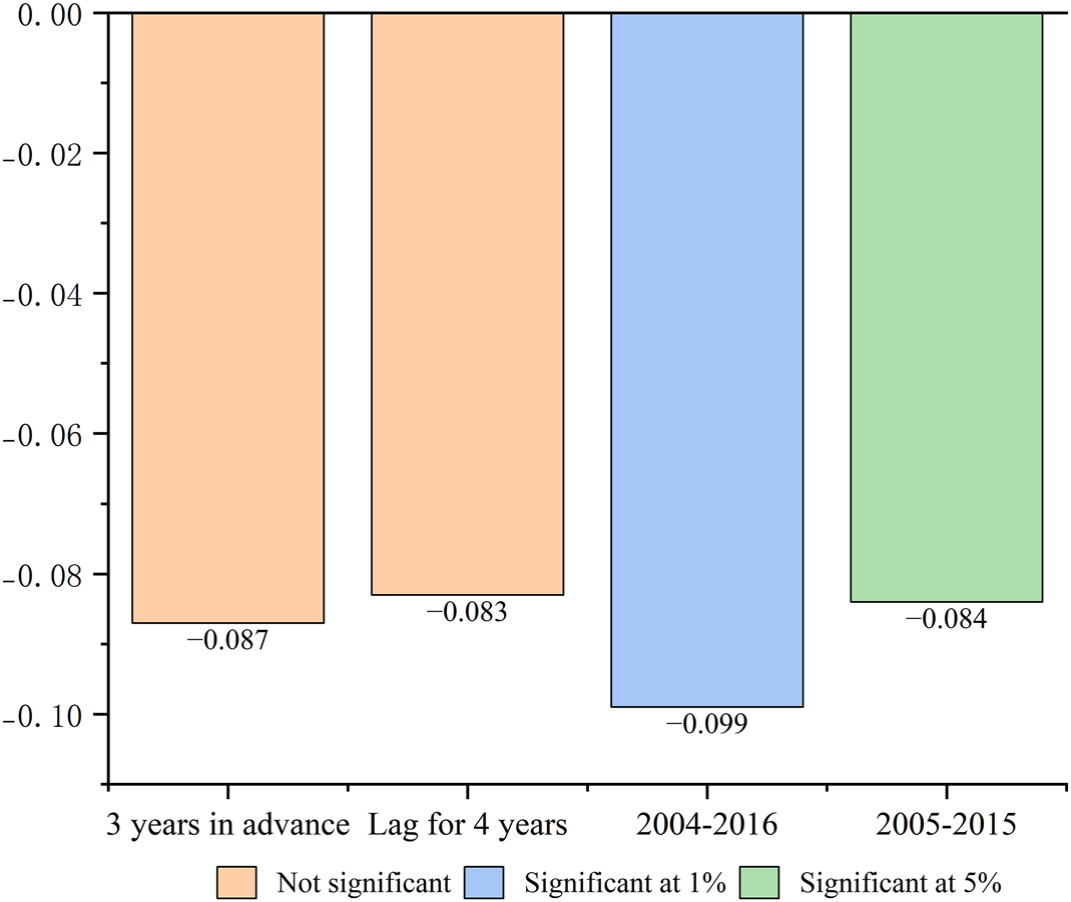
Robustness test. If the regression coefficient assumed by the counterfactual is not significant, and the regression coefficients of different time windows are still significant, it indicates that the robustness is passed.

In Fig. 3, we present the robustness test results of different time windows. The first and second columns are the PSM-DID regression results that we adopt the time placebo method and test the robustness of the model by constructing two counterfactuals, assuming that the ETS policy pilot time is 3 years ahead and 4 years lagged, respectively^32^. The third and fourth columns are the PSM-DID regression results that we change the time window twice for double-testing the robustness of the model, setting the sample data time to 2004–2016 and 2005–2015.

Under the counterfactual assumption, the regression coefficients of the core explanatory variable (*PWQ*) are all negative (*β*_3, 3 years in advance_ =  − 0.087, *β*_3, lag for 4 years_ =  − 0.083), but the results of the policy effect are not significant (*P* > 0.1). We change the time window, the regression coefficients of the core explanatory variable (*PWQ*) are all significantly negative (*β*_3, 2004–2016_ =  − 0.099, *P* < 0.01; *β*_3, 2005–2015_ =  − 0.084, *P* < 0.05). Therefore, this result pass the robustness test.

### Dynamic co-benefits of ETS policy

**Figure 4:**
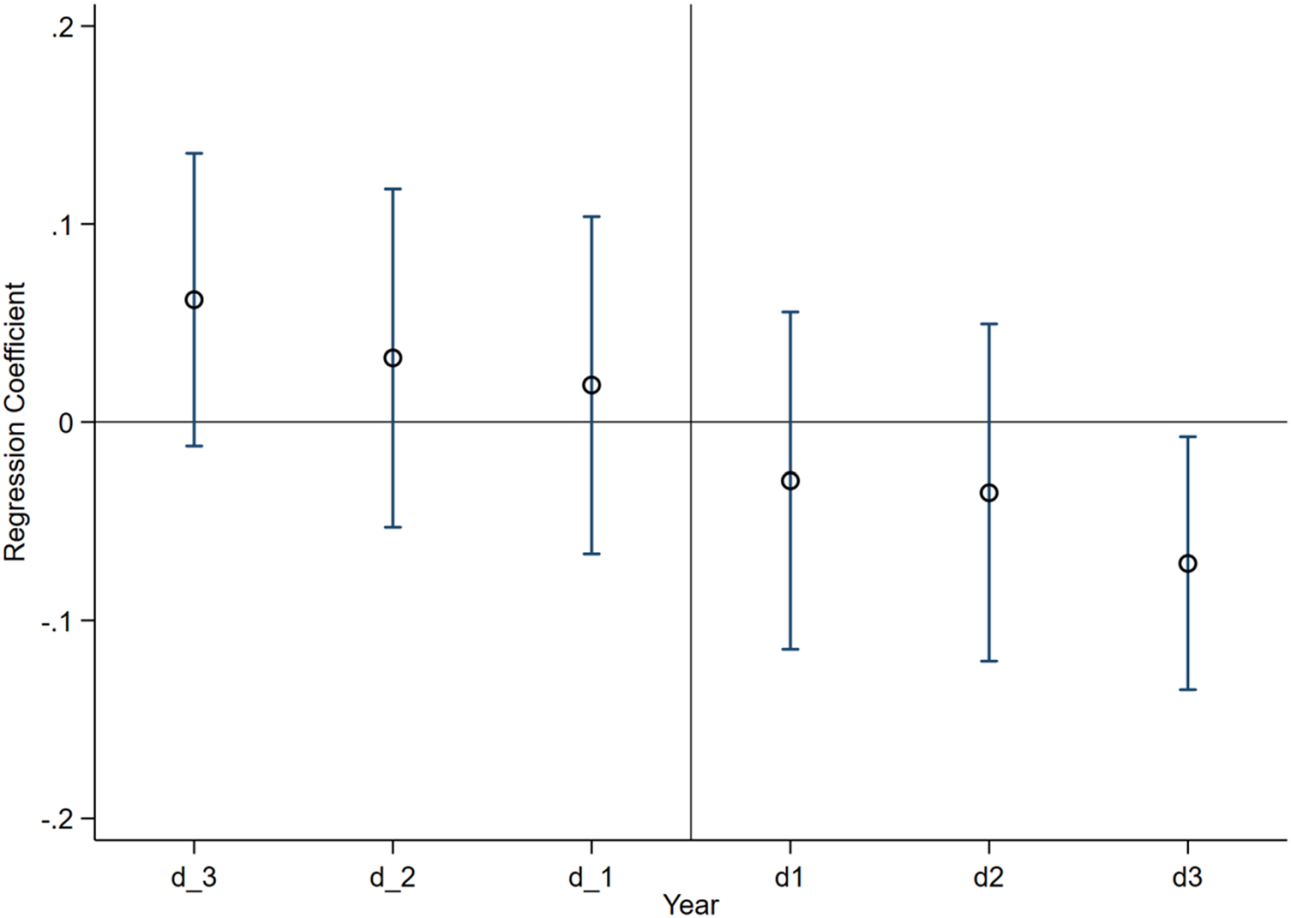
Dynamic co-benefits. (1) d_3: 3 years before the implementation of the policy; d_2: 2 years before the implementation of the policy; d_1: 1 year before the implementation of the policy. (2) d1: 1 year after the implementation of the policy; d2: 2 years after the implementation of the policy; d3: 3 years after the implementation of the policy.

After the implementation of ETS policy, the ETS policy boost energy conservation and emission reduction, and the co-benefits of ETS policy is increasing (Fig. 4). The regression coefficient fluctuates around 0 before the policy is implemented, indicating that the regression coefficient is not significantly different from 0 at the 95% confidence interval. This means that there is no significant difference in the development trend between the experimental group and the control group before the policy is implemented. In addition, after the ETS policy is implemented, the regression coefficient is significantly negative and their absolute values are getting larger and larger. This indicates that the co-benefits of ETS policies are getting greater and greater after the implementation of policies.

### Heterogeneous co-benefits of ETS policy

**Figure 5:**
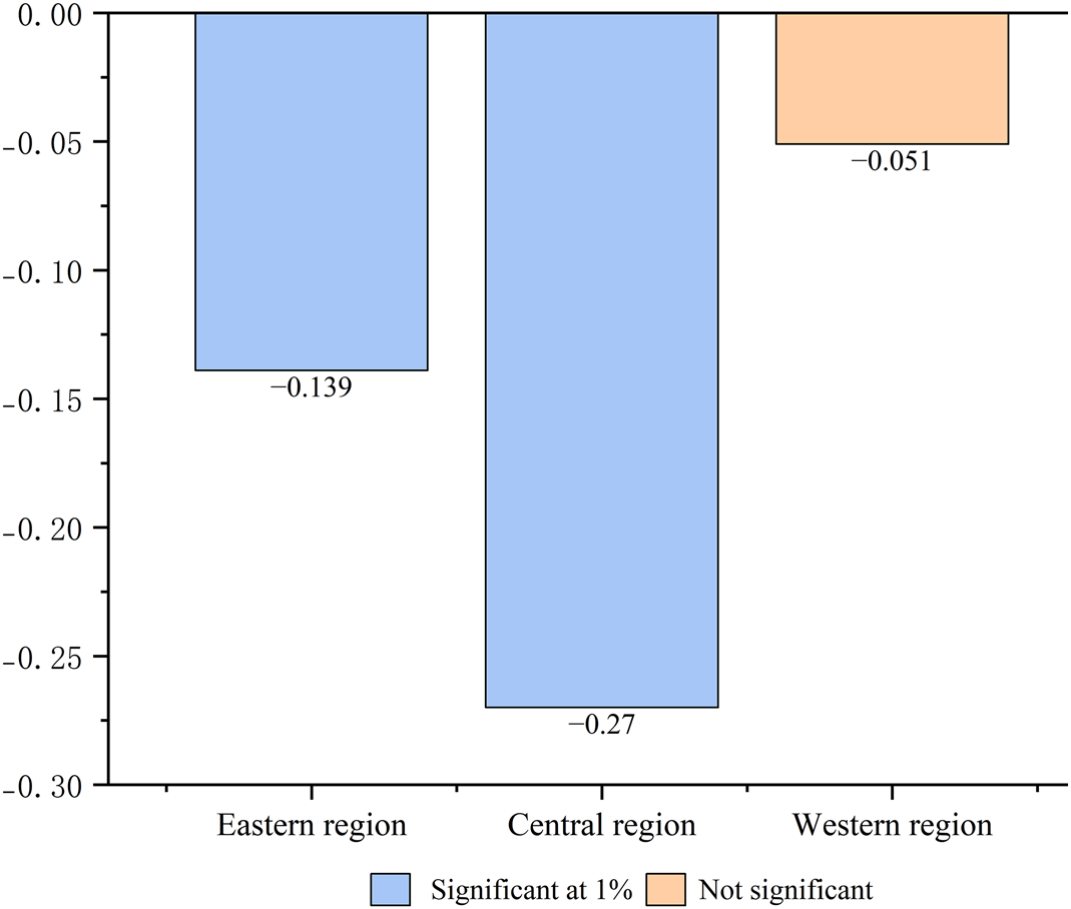
Heterogeneous co-benefits.

We conducted additional analyses to better understand the regional differences in the observed relationship between TES and energy conservation and emission reduction (Fig. 5). Among them, the ETS can significantly promote energy conservation and emission reduction in the eastern and central regions (*β*_3, eastern region_ =  − 0.139, *β*_3, central region_ =  − 0.27, *P* < 0.01). The ETS has no significant effect on energy conservation and emission reduction in the western region (*β*_3, western region_ =  − 0.051, *P* > 0.1).

## Discussion

Under the background of green development in China, understanding the impact of ETS on energy conservation and emission reduction is conducive to jointly responding to global climate change and carrying out global environmental governance. The past experience shows that the realization of environmental governance and energy conservation goals can not only rely on administrative means, but the need also to establish a long-term mechanism. To further establish and improve a series of measures to promote the implementation of the ETS, and to unify and standardize the determination, distribution, trading, and management of emission rights are conducive to promoting the process of energy conservation and emission reduction. It is also fully consistent with the green and low-carbon concept the governments in the world advocate. Therefore, as one of the market incentive environmental regulation methods, the ETS is gradually becoming one of the important means to promote energy conservation and emission reduction and curb global warming.

We find that the implementation of ETS can boost energy conservation and emission reduction, and there is a co-benefit on energy conservation and emission reduction. Although “energy conservation” is closely related to “emission reduction”, it does not mean that there is a simple one-way transmission relationship between the two, that is, “energy conservation” does not necessarily “emission reduction”, and “emission reduction” does not necessarily require “energy conservation”. Considering that there are many types of energy used in economic activities, there are significant differences in the use cost and pollution emission degree of each type of energy, so there is no necessarily positive relationship between energy consumption and CO2 emissions. When enterprise technological innovation is more manifested as efficiency-based technological progress, energy efficiency is generally improved. Under a relatively stable energy structure, “energy saving” will inevitably mean “emission reduction”. In this study, the total ECER index is jointly constructed from the perspectives of “energy saving” and “emission reduction” to explore the synergistic effect of ETS.

The estimation results of PSM-DID verify the effectiveness of the ETS in terms of energy conservation and emission reduction. Appropriate environmental regulations can promote innovative activities among enterprises^33,34^. The effective policy of the ETS has played an innovative effect, and companies have developed new technologies to improve energy efficiency due to innovative activities. Relying on the improvement of energy efficiency, enterprises benefit from the profit of the emission rights of the remaining quotas^18^. Therefore, enterprises finally choose to focus on technological improvement and industrial structure optimization, and the improvement of technological level and industrial structure optimization will eventually reduce energy consumption and reduce pollution emissions.

We find that the effect of ETS on energy conservation and emission reduction in different regions is heterogeneous. The eastern and central regions have a higher degree of marketization and agglomeration of technologies, talents and other elements, and the degree of innovation and technological level have certain advantages, a better policy implementation environment, and regions with a high level of economic development have more resources, which is conducive to improving clean technology and energy conservation and emission reduction. The economic development of the western region depends more on the industry-led industrial structure, which may lead to the lack of motivation to implement ETS, resulting in poor energy conservation and emission reduction. Existing studies have found that the choice of energy consumption and fuel has regional heterogeneity^35–37^, then the regional heterogeneity of China’s energy conservation and emission reduction efficiency is also reasonable^38^.

We adopt different ETS development strategies according to different regional, and increase the intensity of energy conservation and emission reduction. The government departments in the eastern and central regions should continue to increase financial support to promote energy conservation and emission reduction, and give appropriate policy incentives to enterprises that achieve energy conservation and emission reduction targets with high efficiency, appropriately alleviate the tax burden of such enterprises, and stimulate more enthusiasm for enterprises to participate in energy conservation and emission reduction. Government departments in the western region should use multiple channels to effectively absorb advanced technologies in the eastern and central regions, continue to work on the optimization of the industrial structure, encourage industrial enterprises to continue to change the current energy consumption structure, and use technology to improve energy efficiency and reduce energy consumption and pollution emissions, and by continuing to increase its own development conditions, the policy effects of the ETS can be more effectively brought into play.

## Conclusion and implication

### Conclusion

We select the annual data of 30 provinces, municipalities and autonomous region in China (excluding Tibet, Hong Kong, Macao and Taiwan) from 2003 to 2017, and use the DID model and PSM-DID model to study the energy conservation and emission reduction co-benefits of the ETS pilot policy. Then we use, time sensitivity test, and placebo test to verify the robustness of the empirical results, and analyze the dynamic and heterogeneous co-benefits of ETS policy. We draw the following conclusions. First, the ETS pilot policy can significantly promote energy conservation and emission reduction, which has a co-benefit in energy conservation and emission reduction. Second, the impact of the ETS pilot policy on energy conservation and emission reduction in different regions is heterogeneous. The eastern and central regions benefit significantly from the policy effect of ETS, while the western region does not.

### Implication

First, adhere to the principle of government guidance and market leadership, promote the transformation of China’s environmental policy from command and control to market incentive, establish and improve a series of measures to promote the implementation of the ETS, and give full play to the energy conservation and emission reduction potential of the ETS. Secondly, the government should gradually promote the ETS in combination with regional differences such as local industry and endowment structure. Finally, the government and enterprises should adhere to innovation-driven, accelerate the transformation of energy consumption structure from fossil energy to renewable energy and other clean energy, promote the optimization and upgrading of industrial structure, and realize the low-carbon development of industrial system.

### Limitation and future direction

Due to the limitations of data collection, there are still many deficiencies. First of all, the construction of the total index level of energy use and pollution emissions only involves seven variables, and the comprehensive degree of the index is insufficient. Secondly, the system of paid use and trading of emission rights in pilot areas was basically established, and the pilot work was basically completed in 2017. So, this study only examines the impact of the policy on energy conservation and emission reduction in the pilot provinces and cities with emission trading system before 2017, but not after 2017. In addition, this study only studied the impact of ETS policies issued in 2007, and did not focus on the impact of follow-up policies issued from 2011 to 2013, which can be further improved in the future. Finally, this study only briefly analyzes the heterogeneity results of the emission trading system without further analysis of the impact mechanism, which is also something that needs to be further improved in future research.

## Data Availability

The data used in this study are publicly available and were obtained from the National Bureau of Statistics of the People’s Republic of China website (http://www.stats.gov.cn/) and the Chinese Ministry of Environmental Protection’s website (https://www.mee.gov.cn/). The data used in this study is self-retrieval based on public websites, and collated and processed. Therefore, a specific data link can not be provided.
